# Shamanic Kambô Frog Hyponatremic Toxicity Leading to Brain Death: A Case Report

**DOI:** 10.7759/cureus.83963

**Published:** 2025-05-12

**Authors:** Christina Q Tran, Rebecca Houston, Maxwell A Marino, Michael Schiraldi, Dan E Miulli

**Affiliations:** 1 Neurological Surgery, Kaiser Permanente Bernard J. Tyson School of Medicine, Pasadena, USA; 2 Neurosurgery, Desert Regional Medical Center, Palm Springs, USA; 3 Neurological Surgery, Riverside University Health System, Moreno Valley, USA; 4 Neurosurgery, Arrowhead Regional Medical Center, Colton, USA

**Keywords:** brain death critical care, diffuse cerebral edema, hyponatremia, kambo, phyllomedusa bicolor, toxicology

## Abstract

The South American shamanic Kambô ritual involves applying the skin secretions of Phyllomedusa bicolor (giant monkey frog) to superficial burns for purported spiritual and therapeutic benefits. These secretions contain a complex mix of bioactive peptides, such as phyllocaerulein, phyllomedusin, phyllokinin, sauvagine, dermorphins, and deltorphins, that interact with diverse neurotransmitter and hormone receptor systems. Specifically, these peptides engage cholecystokinin (CCK) receptors, neurokinin-1 (NK1) receptors, bradykinin B2 receptors, corticotropin-releasing factor (CRF) receptors, and opioid receptors (μ and δ subtypes), influencing gastrointestinal, cardiovascular, endocrine, and neurologic pathways. While often short-lived, these peptides provoke profound physiological disturbances, including violent emesis, vasodilation, fluid shifts, and antidiuretic hormone (ADH) dysregulation. The combination of these effects with ritualistic overhydration of water can trigger life-threatening hyponatremia, cerebral edema, and ultimately, brain death.

We present the first documented case of brain death associated with Kambô toxicity. A 35-year-old female developed headache, emesis, and unilateral fixed and dilated pupil within hours of a Kambô ritual. Imaging demonstrated diffuse cerebral edema and loss of intracranial blood flow. Laboratory workup revealed severe hyponatremia, likely due to excessive water intake, peptide-induced emesis with sodium loss, and suspected SIADH. Despite aggressive medical management, the patient progressed to brain death.

Kambô rituals are unregulated and increasingly practiced in Western countries despite a lack of medical oversight. The ritual’s unique combination of cutaneous toxin delivery, extreme emesis, and excessive water hydration creates a perfect storm for acute symptomatic hyponatremia and cerebral herniation. Physicians should consider Kambô exposure in patients presenting with acute neurologic decline and dot-like burn patterns. Public awareness, clinician education, and the development of treatment algorithms are critical as the use of this neurotoxic ritual continues to expand.

## Introduction

The Kambô ritual, rooted in the shamanic traditions of the Amazon, involves applying skin secretions of Phyllomedusa bicolor (the giant monkey frog) to superficial burns for spiritual and therapeutic purposes [1]. Although traditionally used to enhance hunting acuity, the ritual has expanded into Western wellness movements and is now promoted as a treatment for depression, anxiety, chronic pain, and autoimmune diseases, despite a lack of experimental evidence [1-3]. The U.S. Food and Drug Administration (FDA) does not regulate Kambô, and no safety guidelines currently govern its use.

During the ritual, practitioners create small burns (“points”) on the skin and apply the frog secretion transdermally [4]. Participants are often encouraged to drink two to four liters of water beforehand, a practice believed to facilitate purging. However, this water-loading, when combined with peptide-induced vomiting, diarrhea, and potential antidiuretic hormone (ADH) dysregulation, poses significant risks of acute dilutional hyponatremia and cerebral edema [3,5].

The bioactive cocktail in Kambô includes phyllocaerulein, phyllomedusin, phyllokinin, sauvagine, dermorphins, and deltorphins, each targeting distinct receptor systems [1]. These peptides stimulate gastrointestinal, cardiovascular, endocrine, and neurologic pathways through cholecystokinin-A (CCK-A), neurokinin-1 (NK1), bradykinin B2, corticotropin-releasing factor 1 (CRF1), and μ-/δ-opioid receptors [1,6,7]. Effects include emesis, vasodilation, increased vascular permeability, sympathetic activation, ADH release, and potential sedation or euphoria. Although potent, these peptides are rapidly metabolized and have short systemic half-lives due to their small size and lack of stabilizing modifications [6].

Adverse effects may manifest within minutes, ranging from flushing and emesis to tachycardia, hallucinations, and neuropsychiatric disturbances [6,8]. Notably, cases of symptomatic hyponatremia, seizures, SIADH, and death have been reported [5,8]. The underlying pathophysiology often reflects a combination of gastrointestinal losses, impaired free water excretion, and water intoxication, creating a cascade that may culminate in cerebral edema and herniation.

In this report, we present the first documented case of brain death attributed to Kambô toxicity. Our aim is to raise awareness of its lethal potential, particularly in the context of acute hyponatremia. As interest in ritualistic medicine grows, physicians must remain vigilant for Kambô-related presentations and understand the underlying mechanisms to initiate timely intervention.

## Case presentation

A 35-year-old female, six months postpartum, with a past medical history of asthma and gastroesophageal reflux disease (GERD), participated in a Kambô ritual for general wellness and spiritual cleansing. According to friends present at the event, she consumed large volumes of water prior to the ritual as part of the facilitator’s prehydration protocol. Approximately five hours later, she complained of severe headaches and experienced recurrent, profuse emesis. She was not known to be on any medications and had no documented psychiatric history. When she began drooling and became increasingly confused and somnolent, friends on the scene called 911. Emergency medical services observed persistent vomiting, progressive somnolence, delayed verbal responses, left-sided weakness, and a right pupil that was fixed and dilated. Despite these alarming findings, the patient remained ambulatory on the gurney with minimal assistance. Initial vital signs revealed normotensive systolic blood pressure, severely decreased oxygen saturation, and elevated blood glucose. En route to the hospital, her condition deteriorated further, with declining responsiveness and hypotension. Upon arrival at the outside hospital, her Glasgow Coma Scale (GCS) was recorded as three, necessitating immediate intubation.

Upon arrival at the outside hospital, the patient was found to be tachycardic, hypotensive, and comatose, necessitating immediate intubation. Laboratory evaluation demonstrated increased white blood cell count, normal hemoglobin, elevated lactate, and decreased sodium, potassium, and chloride levels. Chest radiography revealed bilateral perihilar opacities and a possible left lower lobe infiltrate, suggestive of pulmonary vascular congestion. Despite 100% oxygen, the patient’s oxygen saturation by pulse oximetry remained critically low, suggesting impaired peripheral perfusion. Arterial blood gas analysis status postintubation and on 100% fraction of inspired oxygen showed marked hyperoxia, acidemia, low bicarbonate, and negative base excess, consistent with an uncompensated metabolic acidosis with respiratory compensation (Table 1).

**Table 1:** Vital signs, laboratory findings, and neurological assessment in a patient with acute Kambô toxicity GCS, Glasgow Coma Scale; RA, room air; WBC, white blood cell; pCO₂, partial pressure of carbon dioxide; pO₂, partial pressure of oxygen; HCO₃, bicarbonate; FiO₂, fraction of inspired oxygen

Category	Parameter	Value	Interpretation	Reference Range
Vitals and Neuro Exam	Heart rate	138 bpm	Tachycardia	60-100 bpm
	Blood pressure	60/30 mmHg	Hypotension	90-120/60-80 mmHg
	GCS	3	Comatose	15
	Oxygen saturation	77% on RA	Severe hypoxemia	>94%
Blood Values	WBC count	18 k/mm³	Leukocytosis	4.5-11.0 k/mm³
	Hemoglobin	12.3 g/dL	Normal	12-16 g/dL
Metabolic Panel	Sodium	123 mmol/L	Hyponatremia	135-145 mmol/L
	Potassium	3.1 mmol/L	Hypokalemia	3.5-5.0 mmol/L
	Chloride	92 mmol/L	Mild hypochloremia	96-106 mmol/L
	Lactic acid	6.6 mmol/L	Tissue hypoperfusion	0.5-2.2 mmol/L
Arterial Blood Gas (post-intubation, 100% FiO_2_)	pH	7.2	Acidemia	7.35-7.45
	pCO_2_	31.5 mmHg	Respiratory compensation	35-45 mmHg
	pO_2_	261 mmHg	Hyperoxia	80-100 mmHg
	HCO_3_	12.5 mmol/L	Metabolic acidosis	22-26 mmol/L
	Base excess	-14.0	Uncompensated metabolic acidosis	-2 to +2

**Figure 1:**
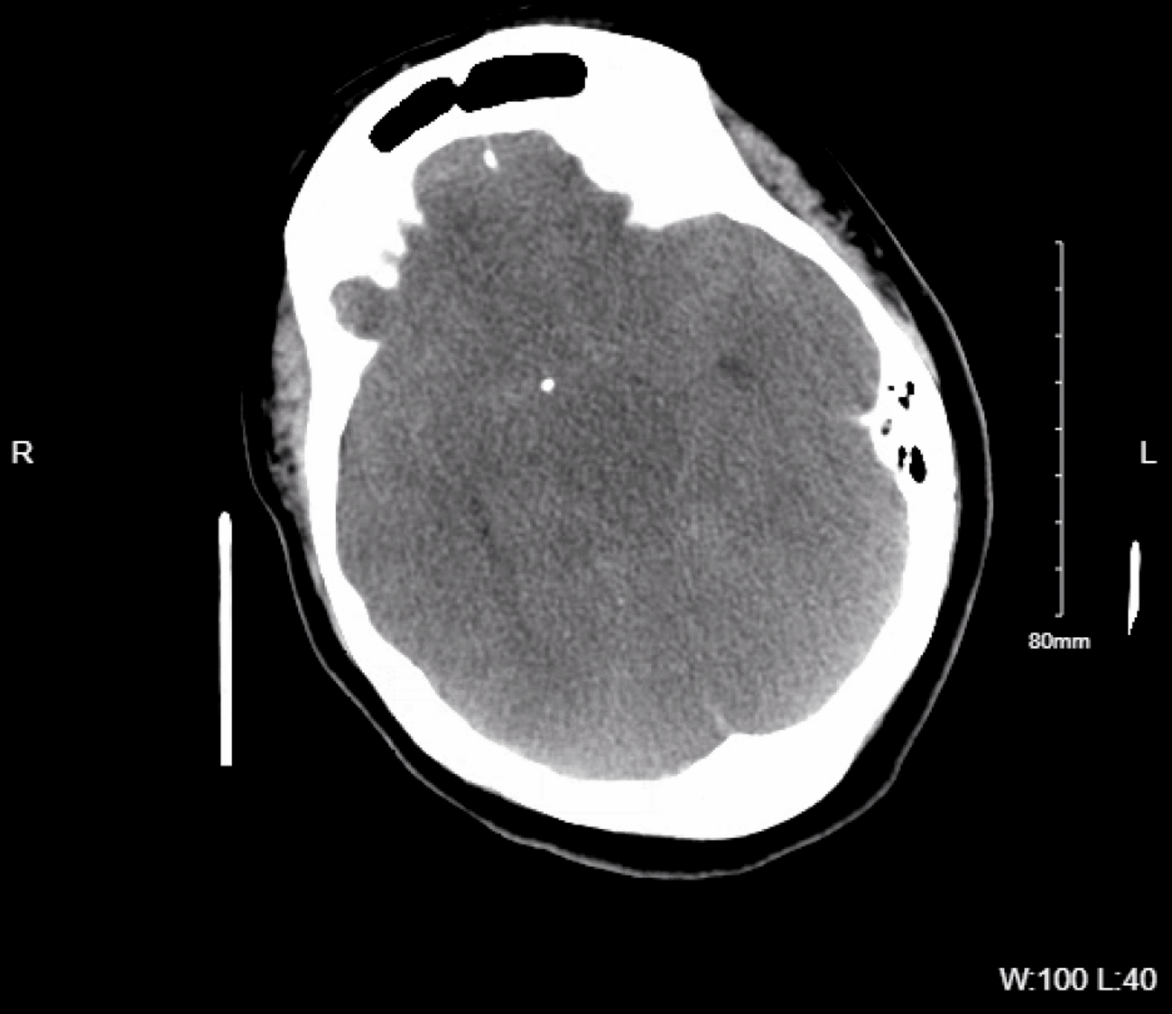
CTH demonstrating diffuse cerebral edema with effacement of basal cisterns and early temporal horns indicative of elevated intracranial pressure CTH, computed tomography of the head

**Figure 2:**
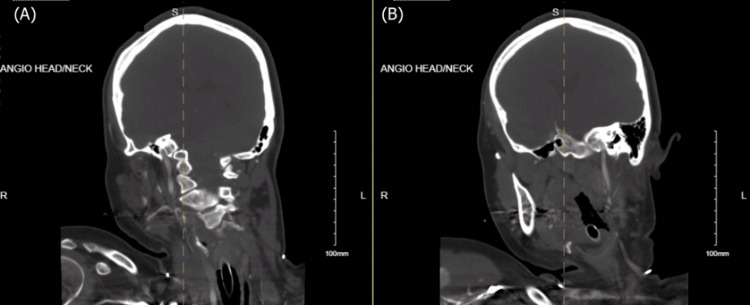
CTA head and neck from outside the hospital demonstrating lack of intracranial contrast with flow beyond the skull base at the (A) right and (B) left internal carotid arteries CTA, computed tomography angiography

The patient was bolused with 3 L of intravenous normal saline (NS), initiated on a norepinephrine drip, and given broad-spectrum antibiotics. Computed tomography angiography (CTA) of the head and neck completed approximately six hours after Kambô administration demonstrated diffuse cerebral edema, loss of basal cisterns, and slight prominence of the temporal horns indicating early hydrocephalus and associated increased intracranial pressure (Figure 1). The scan also demonstrated no contrast in the internal carotid or vertebral arteries beyond the skull base (Figure 2).

The patient was transferred to a tertiary care facility for neuroendovascular and neurosurgical evaluation, given the severity of her presentation. The receiving hospital was a level-one trauma and certified stroke center, and the neuroendovascular and neurosurgery teams were alerted in advance to evaluate for possible thrombectomy, other endovascular interventions, or decompressive craniectomy. She arrived approximately 9.5 hours after Kambô administration. On arrival, the patient remained profoundly hypotensive and hypothermic, with persistently decreased serum sodium and an elevated troponin I level of 1.64 ng/mL. The electrocardiogram showed sinus tachycardia without ischemic changes, and no arrhythmias were noted during initial monitoring.

Train-of-four testing demonstrated four out of four twitches. A neurological exam revealed GCS 3T with bilateral pupils non-reactive and 6 mm in diameter, with absent cough, corneal and gag reflexes, and exhibited no movement to central or peripheral noxious stimuli. Her exam remained unchanged after the administration of escalating vasopressors, 25 grams of 20% mannitol, naloxone 2 mg, 120 mEq of 23.4% hypertonic saline followed by NS, and warming to the normothermic range.

**Figure 3:**
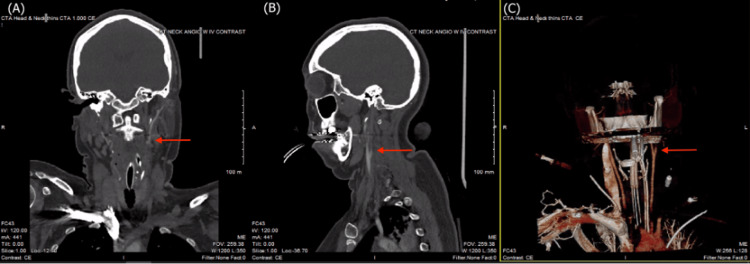
CTA of the head and neck demonstrating absence of flow in the left internal carotid artery approximately 0.6 cm superior to the carotid bifurcation, shown on (A) coronal, (B) sagittal, and (C) 3D reconstruction views CTA, computed tomography angiography

**Figure 4:**
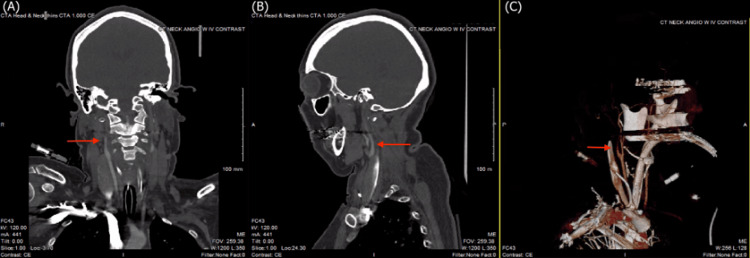
CTA of the head and neck demonstrating absence of flow in the right internal carotid artery approximately 2.6 cm superior to the carotid bifurcation, shown in (A) coronal, (B) sagittal, and (C) 3D reconstruction views CTA, computed tomography angiography

Immediate CTA of the head and neck was consistent with further increased intracranial pressure, as no contrast was present beyond 0.6 cm past the carotid bifurcation on the left, and 2.6 cm on the right (Figure 3 and Figure 4, respectively). Blood flow was demonstrably shunted to the external circulation. Cerebral perfusion testing did not yield any blood flow and was, therefore, unable to produce any imaging for analysis.

Unfortunately, the patient’s clinical and radiographic exams were consistent with catastrophic cerebral injury from prolonged lack of perfusion for many hours secondary to diffuse cerebral edema caused by hyponatremia. As such, no neuroendovascular or neurosurgical intervention was indicated. The patient subsequently had positive brain death exams by two independent physicians and was compassionately extubated.

## Discussion

In 1993, Erspamer et al. determined single peptide concentrations contained in Kambo by bioassay and then correlated the peptides with symptoms of Kambo exposure [7]. Based on this study demonstrating that 10 mg of Kambo are applied to each burn, the peptide content of a single point includes approximately 320 μg phyllocaerulein, 220 μg phyllomedusin, 180 μg phyllokinin, 30 μg sauvagine, 53 μg deltorphin A, and 3 μg dermorphins, the effects of which are discussed herein. With potent action on gastrointestinal smooth muscle and gastric and pancreatic secretions via activation of CCK-A receptors on vagal afferents and enteric tissues, phyllocaerulein is likely the culprit behind the gastrointestinal symptoms of Kambo, which include nausea, vomiting, abdominal discomfort, and the urge to defecate. Phyllokinin and sauvagine are likely to contribute to Kambo’s cardiovascular by activating bradykinin B2 receptors and CRF1 receptors, respectively, leading to peripheral vasodilation, increased vascular permeability, and HPA axis-mediated sympathetic activation, which together produce hypotension and reflex tachycardia. Like all other tachykinins, phyllomedusin has potent effects on blood pressure, gut motility, and salivary and lacrimal gland secretion through substance P-like activity at NK1 receptors, which mediate vasodilation, smooth muscle contraction, and exocrine gland stimulation [7]. Although deltorphin and dermorphin have high affinity for opioid receptors and could theoretically trigger respiratory depression or analgesia via agonism of δ- and μ-opioid receptors in the brainstem and spinal cord, the concentrations in Kambô are likely too low to cause any peripheral effects (Table 2).

**Table 2:** Bioactive peptides in Kambô, their receptor targets, physiological effects, and clinical signs Table Credits: Maxwell Marino, Author. Information adapted from references [1], [2], and [6]. CCK-A, cholecystokinin A; NK1, neurokinin-1; CRF1, corticotropin-releasing factor 1; ADH, antidiuretic hormone

Peptide	Receptor Target	Physiological Action	Clinical Signs and Symptoms
Phyllocaerulein	CCK-A	Cholecystokinin-like; stimulates GI smooth muscle and secretions	Nausea, vomiting, abdominal cramping, diarrhea, the urge to defecate
Phyllomedusin	NK1	Tachykinin; induces vasodilation, smooth muscle contraction, gland secretion	Facial flushing, lacrimation, rhinorrhea, salivation, hypotension
Phyllokinin	Bradykinin B2	Bradykinin analog; increases vascular permeability, promotes vasodilation	Hypotension, flushing, peripheral edema, potential angioedema
Sauvagine	CRF1	CRF-like peptide; stimulates the HPA axis, enhances ADH release, lowers blood pressure	Hypotension, tachycardia, risk of SIADH, and dilutional hyponatremia
Deltorphin A	δ-opioid receptor	High-affinity δ-opioid agonist	Theoretical analgesia, sedation, minimal risk of respiratory depression (low dose)
Dermorphin	μ-opioid receptor	High-affinity μ-opioid agonist	Theoretical analgesia, euphoria; possible respiratory depression
Adenoregulin	Adenosine receptors (experimental)	Adenosine receptor modulator; experimental CNS and cardiac activity	Unknown in humans; may affect sleep, seizure threshold, or cardiac rhythm

A retrospective review of records from 147 subjects who were administered Kambo by a single practitioner in the US performed by Thompson et al. revealed the average number of points administered to be 4.2±2.2 [3]. The most common physical responses to Kambô exposure were facial swelling (70.5%), diaphoresis (53.3%), and bowel movements (45.2%), likely mediated by phyllomedusin-induced NK1 receptor activation, which promotes vasodilation and capillary leak, exocrine gland stimulation, and smooth muscle contraction in the gastrointestinal tract. Participants were predominantly Caucasian (66.7%) and female (68%), with many reporting an autoimmune condition (27.2%) or chronic infectious disease (8.8%), although this may be more reflective of participant affinity for the facilitator, who happens to be a female with an autoimmune disease who gives lectures on the topic.

In 2022, Sacco et al. performed the first systematic review of clinical cases of acute Kambô intoxication from the past decade [8]. Among the 11 analyzed cases, the signs and symptoms of Kambô intoxication included arrhythmias, dyspnea, nausea, vomiting, diarrhea, abdominal pain, weakness, electrolyte imbalances, psychosis, and seizures, mechanistically attributed to peptide-induced autonomic dysregulation, gastrointestinal hyperstimulation via CCK-A and NK1 receptors, vasodilation and fluid shifts via bradykinin B2 receptors, ADH dysregulation via CRF1 receptor activation, and possible central effects of opioid receptor agonists. Electrolyte imbalances that can be triggered by Kambô use include hypokalemia, hypomagnesemia, hypophosphatemia, and most concerningly, hyponatremia, which arise neurophysiologically through peptide-induced activation of gastrointestinal and autonomic pathways, specifically via CCK-A and NK1 receptor stimulation, leading to intense vomiting, diarrhea, and salivary losses, compounded by sauvagine-driven CRF1 receptor activation, which can provoke non-osmotic ADH release and SIADH, impairing renal free water clearance and promoting dilutional electrolyte disturbances [8,9]. Electrolyte loss can be further compounded by concomitant use of other strong purgatives such as ayahuasca or tobacco, which would only act to amplify the violent emesis triggered by Kambo peptides, raise the risk of esophageal rupture due to increased intra-esophageal pressure, and further increase hyponatremia [3,10,11].

This case presentation demonstrates the clinical picture of a patient with Kambô-related severe hyponatremia, diffuse cerebral edema, and subsequent brain death, highlighting the dangerous pathophysiological cascade triggered by Kambô envenomation: phyllocaerulein and phyllomedusin stimulate CCK-A and NK1 receptors, respectively, inducing parasympathetic-driven emesis, diarrhea, and salivary hypersecretion, while sauvagine activates CRF1 receptors, promoting sympathetic-adrenal activation and inappropriate ADH release. This constellation results in massive fluid and sodium loss, impaired renal water excretion, and ritualistic overconsumption of hypotonic fluids, e.g., water, culminating in acute dilutional hyponatremia. The osmotic imbalance drives cytotoxic cerebral edema, increasing intracranial pressure and ultimately leading to herniation and brain death. Five hours after Kambo administration, the patient experienced severe headache, recurrent emesis, increasing somnolence, left-sided weakness, and right pupil fixed and dilated with the patient ambulating on scene. The patient’s unilateral fixed and dilated pupil in the context of preserved consciousness was likely caused by acutely rising intracranial pressure due to worsening cerebral edema, further supported by a deterioration in consciousness on the way to the first hospital. Fixed dilated pupil is considered a grave sign in neurosurgical patients because of its association with poor neurological prognosis and high mortality [12]. Furthermore, fixed dilated pupils accompanied by deteriorating consciousness is a known indicator of acute brainstem compression because components of the reticular activating system are superior to the path of the oculomotor nerve and, therefore, will be primarily affected by downward herniation before the oculomotor nerve is compressed.

Individuals with acute Kambô toxicity may present with hyponatremia due to the combination of ritualistically encouraged consumption of two to four liters of hypotonic water, intended to enhance purging, and the peptide-mediated activation of CCK-A and NK1 receptors by phyllocaerulein and phyllomedusin, which induce intense nausea, vomiting, and gastrointestinal fluid loss; these effects, coupled with sauvagine-induced CRF1 receptor activation, stimulate non-osmotic ADH release, further impairing free water excretion and exacerbating electrolyte imbalances. Acute hyponatremia is especially dangerous because it creates an osmotic gradient between brain tissue and plasma, which promotes the movement of water into brain cells, causing cerebral edema and associated neurological deficits. Within the first three hours, the brain adapts to hyponatremia by moving electrolytes from inside the cells to the extracellular compartment [9]. However, due to the limited nature of electrolyte depletion in the brain, approximately 18% of its total ion content, once the mechanisms of electrolyte loss are exhausted, prolonged severe hyponatremia will inevitably lead to cerebral edema. Neurological sequelae like seizures or coma and death are also more likely if the rate of sodium drop exceeds 1 mEq/L per hour [8,9].

The illustrated patient had acute severe hyponatremia, as indicated by serum sodium 123 mmol/L. Decreasing sodium from low normal 135 to 123 might be tolerated if occurring over 12 hours but far less endured over five hours as was this case. Prolonged hyponatremia led to subsequent diffuse cerebral edema, early hydrocephalus, and increased intracranial pressure as confirmed by loss of basal cisterns and slight prominence of the temporal horns on head CT (Figure 2). The chest x-ray findings of perihilar opacities and opacity in the left lung base indicate pulmonary vascular congestion. This is explained by the swelling of brain parenchyma, which would compress the vasculature, resulting in cerebral hypoxia and subsequent neurogenic pulmonary edema, which further decreases oxygen delivery to the brain [9]. The patient then experienced brain death, as supported by CTA demonstrating no contrast in the internal carotid or vertebral arteries beyond the skull base (Figure 3) in addition to being officially confirmed by two different physicians.

This is the first case of brain death secondary to acute Kambo intoxication, in addition to being the first Kambo-related death in the US to be recorded in the scientific literature. While Kambo is being studied for potential medical applications due to its potent bioactive peptides, those studies are still in their very early stages and currently, there is no FDA-approved use for Kambo in the US. While other countries including Australia and Brazil have banned Kambo, it still poses a significant threat as long as both the quality of Kambo and the practitioners themselves remain unregulated, which is currently the case in the US. With the claims of Kambo practitioners largely going unchallenged, in tandem with the sporadic reporting of acute adverse events caused by Kambo use, it leaves the general public with a misinformed perception regarding the dangers of participating in Kambo rituals. This case is presented to educate medical professionals and the general public on the extreme risks associated with exposing oneself to animal toxins containing bioactive peptides at uncontrolled concentrations, during unregulated Kambo ceremonies performed by practitioners who encourage participants to drink excessive amounts of water, unlike their Amazonian counterparts who drink frog tea, unaware that they are putting people they are supposed to be healing directly in harm’s way.

In regard to previously reported cases, one paper has reported Kambô-related hyponatremia, and another described the first death associated with Kambô use; however, in the latter, the subject’s preexisting conditions made it unclear whether the death was directly attributable to Kambô intoxication. Leban et al. reported a separate case involving a 44-year-old woman who developed symptomatic hyponatremia due to SIADH after drinking six liters of water during a Kambô ritual. She experienced nausea, vomiting, weakness, confusion, lethargy, seizure, short-term memory loss, and decreased consciousness. Notably, that patient underwent successful sodium correction, fully recovered, and was discharged from the hospital [13]. This stands in contrast to our index case, in which the 35-year-old female patient deteriorated despite aggressive management and progressed to brain death within hours of presentation.

In 2017, Aquila et al. reported the case of a 42-year-old man with a medical history of obesity, chronic Kambo use, moderate coronary artery disease (CAD), and left ventricular hypertrophy (LVH), who was found deceased near a box of Kambo sticks approximately half an hour after self-envenomation [14]. Physical examination revealed nine small burn marks on his left arm consistent with Kambo use, although information about duration or frequency of use was unavailable. Toxicological analysis of the subject’s blood isolated deltorphin A at concentrations of 5.00 μg/mL via protein precipitation followed by liquid chromatography with tandem mass spectrometry, a level that, while not well-characterized in human toxicity thresholds, suggests systemic absorption at pharmacologically active levels, particularly given deltorphin’s high potency at δ-opioid receptors and the potential for synergistic cardiovascular or central effects when combined with other Kambô peptides [14,15]. Although the case by Aquila and colleagues was the first Kambô-related death reported in the literature, the authors could not exclude the possibility of the death being unrelated to Kambô use. They proposed two potential explanations for the man’s death: reduced myocardial perfusion secondary to Kambô’s hypotensive effects or Kambô-related tachycardia triggering a cardiac arrhythmia in the setting of LVH, both of which could plausibly be attributed to phyllokinin-mediated bradykinin B2 receptor activation, causing systemic vasodilation and hypotension, and sauvagine-induced CRF1 receptor stimulation, contributing to sympathetic overactivity and tachycardia.

In patients with suspected Kambo toxicity, laboratory evaluation should include a panel of routine electrolytes and the collection of calcium, magnesium, phosphate, and transaminase levels [16]. In patients with existing cardiovascular compromise, particular attention should be paid to electrolyte abnormalities that could exacerbate arrhythmias [17]. Initial management should include isotonic fluid resuscitation (e.g., 0.9% NS) to restore intravascular volume, particularly in hypotensive patients. This may then be followed by hypertonic saline (e.g., 3% or 23.4%) in cases of symptomatic or severe hyponatremia to mitigate the risk of cerebral edema and herniation. Correction of other electrolyte abnormalities, especially hypokalemia and hypomagnesemia, should also be addressed, along with symptomatic treatment of nausea and vomiting to prevent further derangements in fluid balance [18].

**Figure 5:**
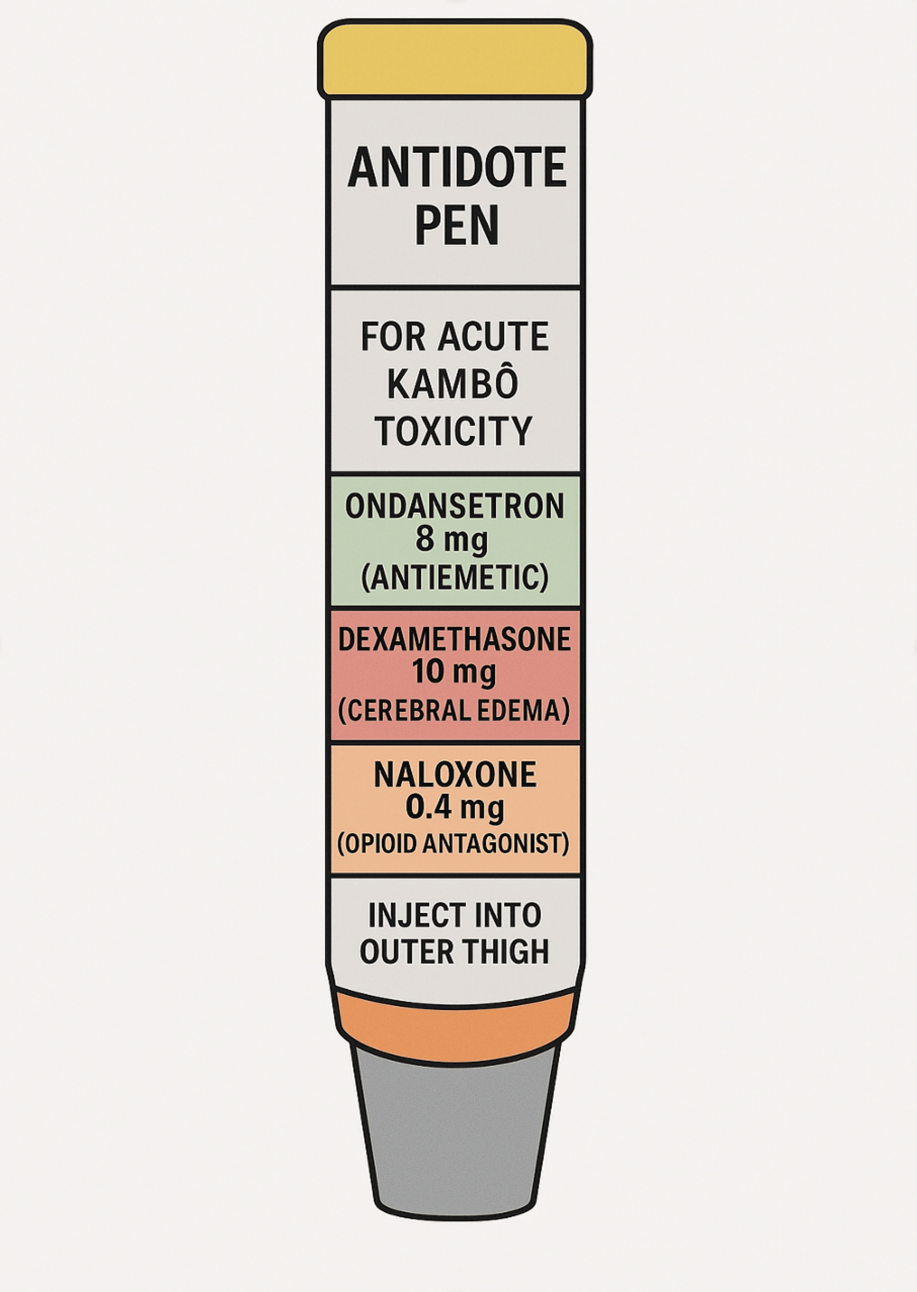
Conceptual schematic of a theoretical Kambô “antidote pen,” designed for prehospital administration in cases of suspected Kambô toxicity. Proposed contents include ondansetron for emesis control, dexamethasone to mitigate cerebral edema, and naloxone as a safety measure against possible opioid-related respiratory depression Image Credits: Maxwell Marino, Author.

Given the growing prevalence of Kambô use in non-traditional settings and the significant risk of morbidity and mortality associated with its administration, we propose a theoretical emergency intervention, an “antidote pen”, modeled after the epinephrine autoinjector, to be deployed in high-risk or remote environments (Figure 5). An internet-based survey conducted in 2021 included 386 participants, predominantly from Western countries, with a mean age of 38.1 years [19]. The study found that 67.9% of respondents had prior experience with ayahuasca, indicating a significant overlap with psychedelic communities. This data underscores the expansion of Kambô practices beyond indigenous Amazonian contexts into urban and alternative medicine circles in Western countries since the early 2000s. The principal danger in acute Kambô toxicity arises from severe hyponatremia-induced cerebral edema, primarily mediated by excessive secretion of ADH/vasopressin and CRF, which together impair renal water excretion and promote intracellular cerebral water shifts. These neuroendocrine pathways are stimulated by Kambô peptides such as sauvagine, a CRF analog, in addition to physiologic stress and vomiting-induced hypovolemia. The resulting osmotic gradient facilitates rapid water influx into astrocytes, leading to cytotoxic cerebral edema, increased intracranial pressure, and potentially, brain herniation.

The antidote pen itself would not contain hypertonic saline, as its high volume and vascular irritation make intramuscular (IM) or subcutaneous (SQ) delivery impractical. Instead, hypertonic saline (e.g., 3% NaCl) should remain a mainstay of prehospital or emergency department intravenous therapy when Kambô toxicity is suspected. The pen-like delivery system would focus on supportive agents: ondansetron (8 mg IM) to control intense nausea and vomiting mediated by phyllocaerulein and phyllomedusin; dexamethasone (10 mg IM) to reduce cerebral edema and blunt systemic inflammatory or allergic responses; and naloxone (0.4-2 mg IM) as a precautionary measure in cases of poly-substance use or respiratory depression potentially triggered by dermorphins or deltorphins. While still theoretical, such a field-deployable antidote pen-like delivery system could offer critical stabilization during the window before definitive medical care becomes available. The International Association of Kambô Practitioners (IAKP) has trained over 400 practitioners since its founding in 2014, offering a two-week training course [20]. With no regulatory body in the US overseeing Kambô use, the responsibility falls on prospective participants to consult with a physician regarding the risks of Kambo, screen for contraindications, and evaluate practitioner credibility on the IAKP website [21]. The rise of the global alternative health movement has propagated the spread of many cultural rituals far from their places of origin, which leaves significant risk for some people to undertake such practices without prior awareness of possible risks, necessary preparations, or traditional dosages.

## Conclusions

Kambô rituals represent an emerging public health challenge, particularly as their practice expands into unregulated, non-clinical settings far removed from their cultural origins. The combination of bioactive peptide exposure and ritualistic water-loading can result in profound electrolyte disturbances, culminating in cerebral edema, increased intracranial pressure, and brain herniation. This case demonstrates the devastating neurological consequences that can follow even a single Kambô exposure. From a neurosurgical perspective, early recognition of fixed and dilated pupils, rapid neurologic decline, and radiographic signs of global cerebral edema are essential for timely triage and prognostication. However, definitive intervention may be precluded by the irreversible nature of osmotic brain injury in acute hyponatremia. Thus, prevention and education remain the most effective tools to mitigate risk.

Clinicians should be attuned to Kambô toxicity in patients with gastrointestinal and neurologic symptoms, especially in the presence of distinctive burn marks. Diagnosis is primarily clinical, and delays in supportive care, especially sodium correction, can be fatal. Public health agencies and medical providers must collaborate to improve awareness, develop prehospital treatment protocols, and address the broader implications of alternative medicine practices that carry life-threatening risks.
